# Publisher Correction: Frugal alignment-free identification of FLT3-internal tandem duplications with FiLT3r

**DOI:** 10.1186/s12859-022-05079-x

**Published:** 2022-12-09

**Authors:** Augustin Boudry, Sasha Darmon, Nicolas Duployez, Martin Figeac, Sandrine Geffroy, Maxime Bucci, Karine Celli-Lebras, Matthieu Duchmann, Romane Joudinaud, Laurène Fenwarth, Olivier Nibourel, Laure Goursaud, Raphael Itzykson, Hervé Dombret, Mathilde Hunault, Claude Preudhomme, Mikaël Salson

**Affiliations:** 1 Hematology Laboratory, Centre de Biologie Pathologie Génétique, CHU Lille, Lille, France; 2 Univ. Lille, CNRS, Centrale Lille, UMR 9189 CRIStAL, F-59000 Lille, France; 3 ENS Lyon, Lyon, France; 4 U1277 Cancer Heterogeneity Plasticity and Resistance to Therapies (CANTHER), University of Lille, INSERM, Lille, France; 5 Hematology Department, CHU LILLE, Lille, France; 6 Univ. Lille, CNRS, Inserm, CHU Lille, Institut Pasteur de Lille, US 41 - UMS 2014 - PLBS, F-59000 Lille, France; 7 Department of Hematology, Saint Louis Hospital, Assistance Publique-Hôpitaux de Paris (APHP), Paris, France; 8 INSERM/CNRS UMR 944/7212, Saint-Louis Research Institute, Paris Diderot University, Paris, France; 9 Univ Angers, Université de Nantes, CHU Angers, Inserm, CNRS, CRCI2NA, SFR ICAT, F-49000 Angers, France

**Correction to: BMC Bioinformatics (2022) 23:448** https://doi.org/10.1186/s12859-022-04983-6

Following publication of the original article [1], the authors identified that the files in Additional file 1 were not separately listed in additional files. This has been corrected since then.

The original article [1] has been corrected.

## Supplementary Information

**Additional file 1.** supp file 1: filt3r-supp.pdf.

**Additional file 2.** supp file 2: results_sra.xlsx.

**Additional file 3.** supp file 3: accessions.csv.

**Additional file 4.** supp file 4: results-different-k.xlsx.

**Additional file 5.** supp file 5: results-small-k.xlsx.

**Additional file 6.** supp file 6: results-different-perc.xlsx.

**Additional file 7.** supp file 7: results-subsamples.xlsx.

**Additional file 8.** supp file 8: genescan_SRR15006459.pdf.

**Additional file 9.** supp file 9: genescan_SRR15006372.pdf.

**Additional file 10.** supp file 10: accessions-CCLE.csv.

**Additional file 11.** supp file 11: results_simulated_150bp.xlsx.

**Additional file 12.** supp file 12: results_simulated_250bp_hq.xlsx.

**Additional file 13.** supp file 13: results_simulated_250bp_lc.xlsx.

**Additional file 14.** supp file 14: results_simulated_250bp_lq.xlsx.

**Additional file 15.** supp file 15: results_simulated_250bp.xls.
