# Supplementary figures and images for: Frugal alignment-free identification of FLT3-internal tandem duplications with FiLT3r
Source: BMC Bioinformatics. 2022 Oct 28;23:448. doi: 10.1186/s12859-022-04983-6 (PMC9617311; doi:10.1186/s12859-022-04983-6)

| Sample File | Sample Name | OS | SQ |
|-------------|-------------|----|----|
|             |             |    |    |

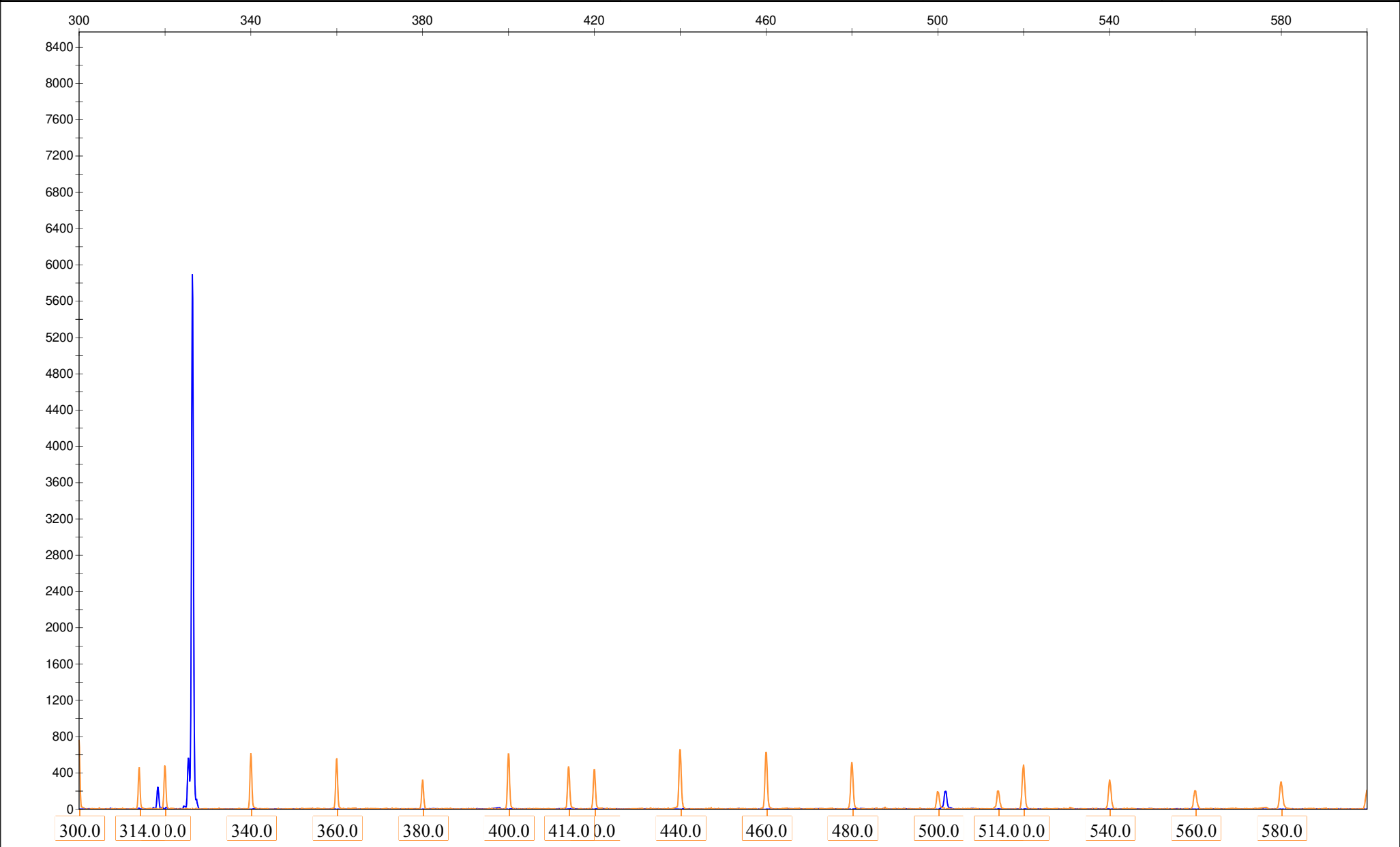

Supplement: Supplementary file 8 — Additional file 8. supp file 8: genescan_SRR15006459.pdf. [file 12859_2022_4983_MOESM8_ESM.pdf]

| Sample File | Sample Name | OS | SQ |
|-------------|-------------|----|----|
|             |             |    |    |

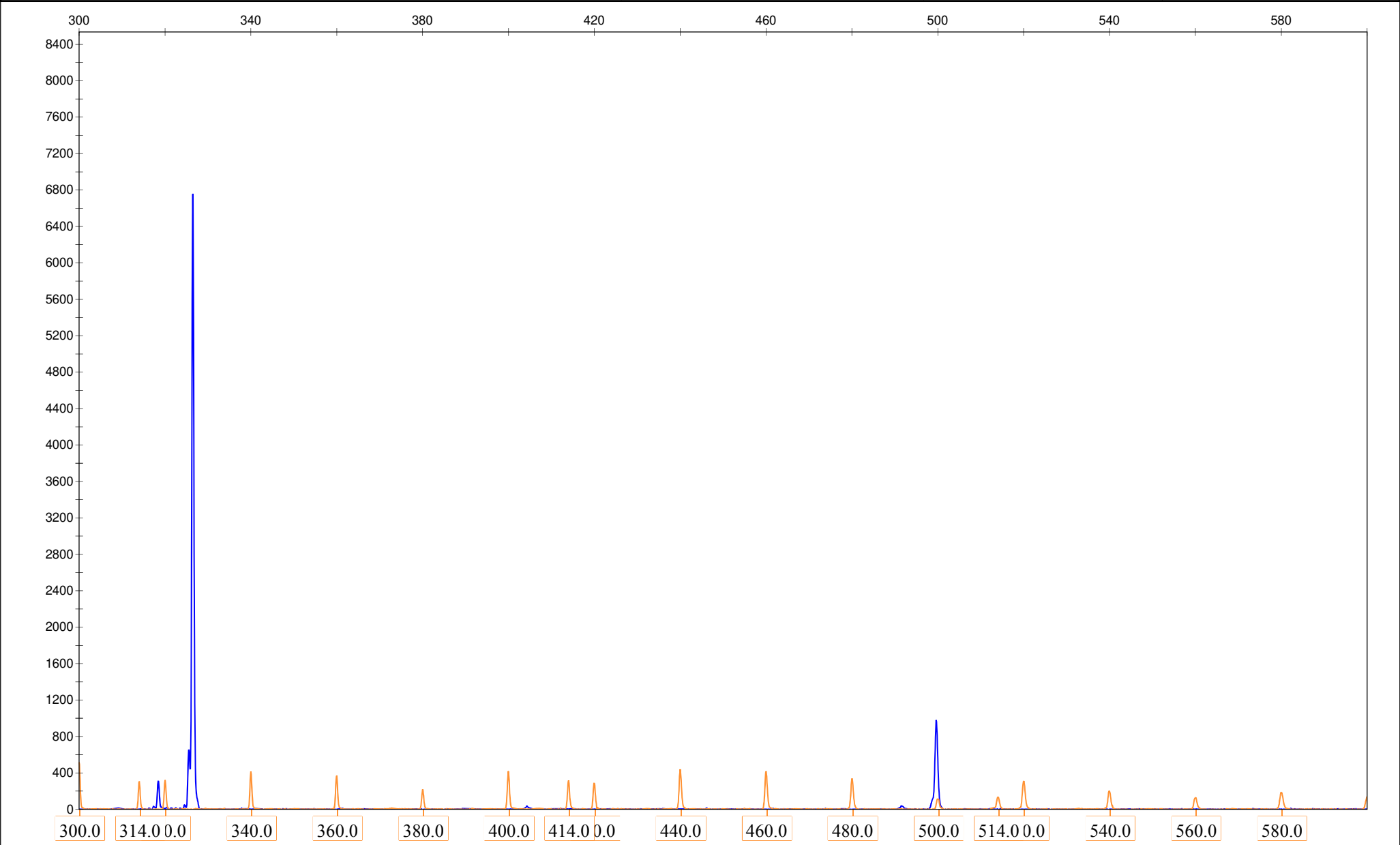

Wed Jul 07,2021 03:20PM, CEST Printed by: gm Page 3 of

Supplement: Supplementary file 9 — Additional file 9. supp file 9: genescan_SRR15006372.pdf. [file 12859_2022_4983_MOESM9_ESM.pdf]
